# Guanidinoacetic acid in human nutrition: Beyond creatine synthesis

**DOI:** 10.1002/fsn3.3201

**Published:** 2023-01-11

**Authors:** Sergej M. Ostojic, Jagoda Jorga

**Affiliations:** ^1^ Applied Bioenergetics Lab, Faculty of Sport and Physical Education University of Novi Sad Novi Sad Serbia; ^2^ Department of Nutrition and Public Health University of Agder Kristiansand Norway; ^3^ Faculty of Health Sciences University of Pécs Pécs Hungary; ^4^ Department of Hygiene and Medical Ecology, School of Medicine University of Belgrade Beograd Serbia

**Keywords:** arginine, creatine, guanidinoacetic acid, insulin, NAFLD, taste

## Abstract

Guanidinoacetic acid (GAA) is a nutrient that has been used in human nutrition since the early 1950s. Recommended for its role in creatine biosynthesis, GAA demonstrated beneficial energy‐boosting effects in various clinical conditions. Dietary GAA has also been suggested to trigger several creatine‐independent mechanisms. Besides acting as a direct precursor of high‐energy phosphagen creatine, dietary GAA is suggested to reduce blood glucose concentration by acting as an insulinotropic food compound, spare amino acid arginine for other metabolic purposes (including protein synthesis), modulate taste, and perhaps alter methylation and fat deposition in various organs including the liver. GAA as a food component can have several important metabolic roles beyond creatine biosynthesis; future studies are highly warranted to address GAA overall role in human nutrition.

## Graphical abstract

Dietary exposure to guanidinoacetic acid (GAA) has been suggested to trigger several creatine‐unconnected mechanisms. Besides acting as a direct precursor of high‐energy phosphagen creatine, dietary GAA could reduce blood glucose concentration by acting as an insulinotropic food compound, spare amino acid arginine for other metabolic purposes (including protein synthesis), modulate taste, and perhaps alter methylation and fat deposition in various organs including the liver. GAA as a food component can have several important metabolic roles beyond creatine biosynthesis; future studies are highly warranted to address GAA overall role in human nutrition.

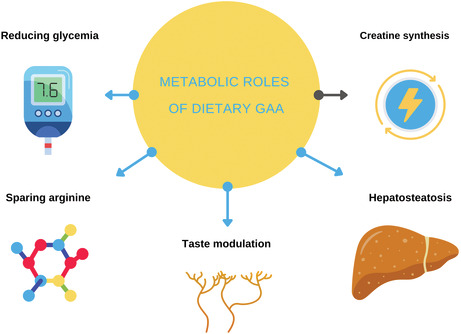

## 1 BACKGROUND

Guanidinoacetic acid (GAA; also known as glycocyamine, molecular formula: C_3_H_7_N_3_O_2_) is a naturally occurring member of the class of organic compounds known as alpha‐amino acids. GAA is an *N*‐amidino derivative of glycine and *L*‐arginine, and contains guanidino moiety that can play a significant role in the interaction with various enzymes or receptors (Kubik & Mungalpara, 2017). GAA is synthesized in the human body but could also be provided by the animal‐ and plant‐based foods, and nutritional supplements. This guanidino compound is predominantly involved in human bioenergetics, acting as a direct precursor of creatine, the primary high‐energy phosphate storage molecule. GAA role in creatine biosynthesis and upholding energy metabolism has been recognized and exploited in human nutrition for over 70 years (for a detailed review, see Ostojic, 2016, 2017). Still, its use as a food component might comprise several non‐creatine‐related functions. This review paper summarizes key aspects of GAA metabolism, and overviews several alternative roles of dietary GAA in clinical and experimental nutrition, including glucose regulation, arginine sparing, and taste modulation.

## 2 GAA METABOLISM

GAA is mainly synthesized in the human kidney and pancreas (also the brain, liver, endocrine tissues, and skin) from glycine and *L‐*arginine, two conditionally essential amino acids. This simple transamination reaction is catalyzed by the enzyme *L*‐arginine: glycine amidinotransferase (AGAT). After this initial step, GAA is delivered to the liver, where the methyl group is transferred from *S*‐adenosyl‐*L*‐methionine (SAMe) to GAA by the action of guanidinoacetate *N*‐methyltransferase (GAMT) to produce creatine. Approximately one gram of creatine is synthesized per day via this pathway, perhaps utilizing most of the GAA available in the human body. The reference serum and urinary GAA levels are 2.3 μmol/L (mixed), 31.2 mmol/mol creatinine (male), and 53.1 (female) mmol/mol creatinine in healthy humans aged over 15 years, respectively (Joncquel‐Chevalier Curt et al., 2013), with GAA also detected in human milk, saliva, and cerebrospinal liquor. GAA synthesis and utilization could be compromised in various pathological conditions, with creatine deficiency syndromes being most featured (for a detailed review, see Ostojic, Ratgeber, et al., 2020).

## 3 DIETARY INTAKE OF GAA

**FIGURE 1:**
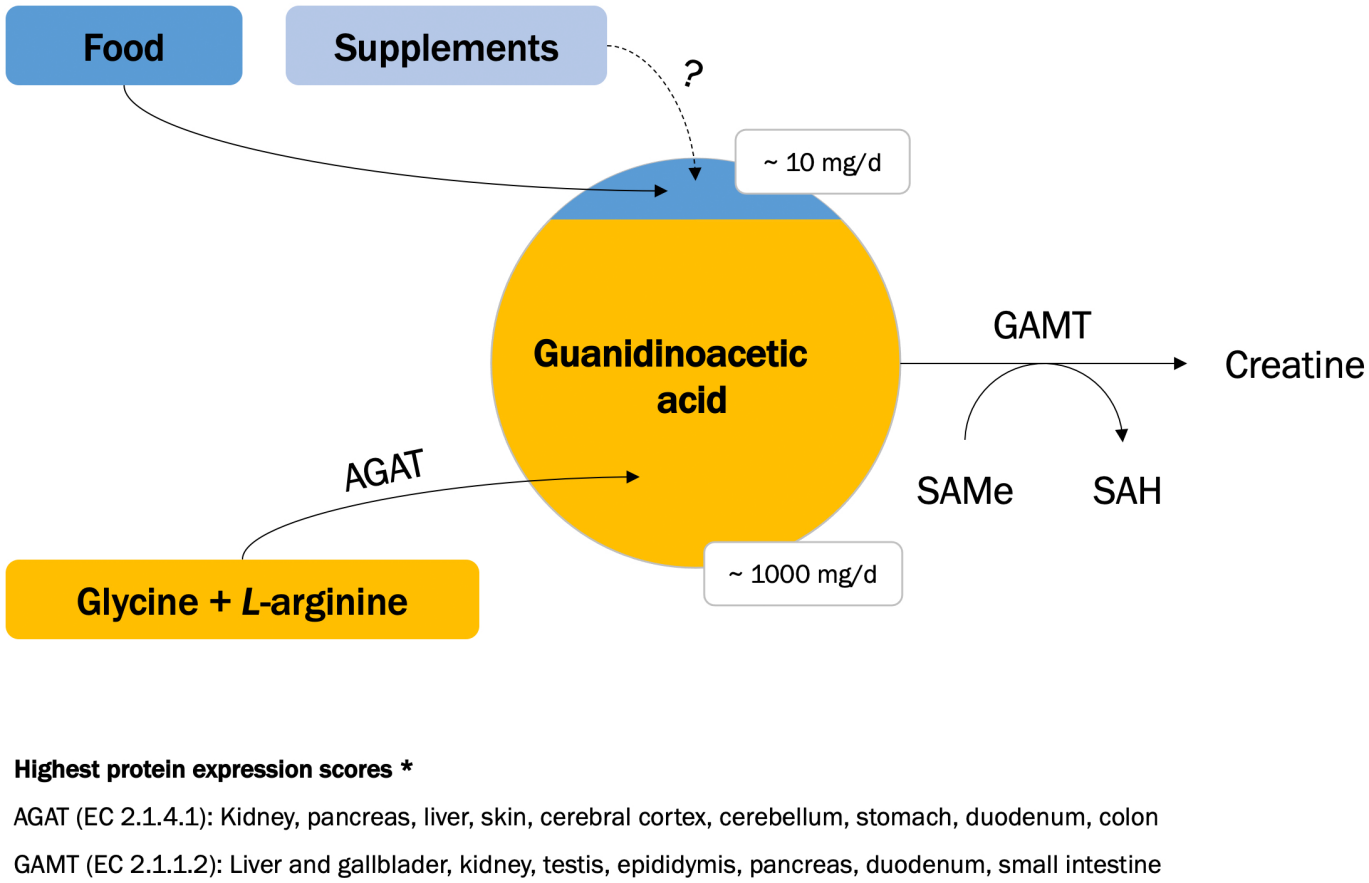
The framework of GAA intake, metabolism, and utilization. Abbreviations: AGAT, *L*‐arginine: Glycine amidinotransferase; GAMT, guanidinoacetate *N*‐methyltransferase; SAMe, *S*‐adenosyl‐*L*‐methionine; SAH, *S*‐adenosyl‐*L*‐homocysteine. The estimation for GAA exposure is presented as mean values for an average adult. * Protein expression scores are revealed from The Human Protein Atlas (https://www.proteinatlas.org/).

GAA is available from various food sources, including red meat and poultry, milk, and a few plant‐based foods (Ostojic, 2022). Meat‐based products contain the highest relative amount of GAA (~50 mg per kg), followed by a dairy group (~0.3 mg per kg), and plant‐based foods (~1 μg per kg), suggesting relatively low dietary exposure to exogenous GAA in omnivores, and even lower in vegans/vegetarians. We recently confirmed this hypothesis in a population‐based study in U.S. adults, where the mean dietary intake of GAA was ~10 mg per day (Ostojic et al., 2022), with men consuming more GAA than women (12 vs. 8 mg/day). A small amount of GAA provided via regular diet implies that de novo synthesis of GAA from glycine and *L*‐arginine likely provides a larger part of this compound (Figure 1). GAA could also be obtained via dietary supplements, with several products available on the international market containing up to 1.0 g of GAA per single serving. A typical daily dose of supplemental GAA is ~66 mg per kg of body weight (Borsook & Borsook, 1951a), usually combined with other compounds (e.g., betaine, choline, creatine, B vitamins). No data are currently available concerning the daily needs and replacement of GAA.

## 4 GAA HOMEOSTATIC VARIATION

Recent studies demonstrate changes in GAA biodynamics due to various acute and chronic physiological stimuli, including fasting, exercise, and aging. GAA decreased dramatically in all organs within 24 h after starvation, with low levels maintained for up to 96 h, except for the brain and plasma (Shindo et al., 1986). Fasting appears to reduce AGAT activity while *L*‐arginine and glycine were not regulatory factors of GAA level in starved conditions (Shindo et al., 1986); these results suggest that AGAT is inhibited in starved conditions by some unknown factors. Acute exercise also affects serum GAA levels, with a single session of exhaustive exercise markedly decreasing circulating levels of GAA in healthy men and women by up to 49.8% (Stajer et al., 2016). Low GAA availability could be due to an exercise‐induced reduction in GAA production in the kidney, increased GAA utilization to creatine in the liver, or both. Interestingly, higher circulating levels of GAA were accompanied by advanced age in healthy women (Olah et al., 2019), implying altered homeostasis between GAA synthesis, utilization, and/or elimination in this population.

## 5 INSULINOTROPIC EFFECTS OF GAA

A seminal clinical study from Caltech was arguably the first to demonstrate a glucose‐lowering effect of dietary GAA. Borsook and Borsook (1951b) reported a moderate drop in blood glucose levels in patients receiving one gram of GAA per day during a period of 6 to 10 months, with glucose tolerance increased in both diabetic and non‐diabetic patients. A slight downward trend in blood glucose is confirmed in another historical trial (no absolute changes reported), with patients suffering from arthritis receiving five grams of GAA per day for up to 42 days (Higgins et al., 1952). Several animal studies demonstrated that GAA could stimulate insulin secretion and/or reduce circulating glucose levels, with the insulinotropic effect of GAA superior to other amino acid derivatives and guanidines (Alsever et al., 1970; Aynsley‐Green & Alberti, 1974; Meglasson et al., 1993; Zhang et al., 2019). Although a possible clinical potential of GAA‐induced insulin stimulation is yet to be revealed, GAA appears to share transport kinetics with its analog beta‐guanidinopropionic acid, an antihyperglycemic therapeutics (Metzner et al., 2009). Interestingly, serum, urinary, and renal cortex GAA levels are affected by streptozotocin‐induced diabetes (Kiyatake, 1994), with concentrations returned to the control levels after insulin treatment. This suggests a possible bidirectional connection between GAA and insulin biodynamics, and the rather complex role of GAA in glucose homeostasis that might also involve insulin‐like growth factor I (Liu et al., 2021; Michiels et al., 2012) and glucagon (Marco et al., 1976). Still, a GAA‐driven reduction in blood glucose might be relevant only in individuals with impaired glucose metabolism since dietary GAA appears ineffective in altering serum insulin levels in healthy men and women (Ostojic et al., 2018).

## 6 ARGININE SPARING

Arginine is a conditionally essential amino acid that plays many important roles in the human body, from cell division and gene expression, to the synthesis of proteins, urea, nitric oxide, creatine, and other biologically important compounds (Morris Jr., 2006). Theoretically, being a direct precursor of creatine, GAA can spare arginine for creatine synthesis, and save it for other biological functions. Several feeding studies have shown that GAA added to low‐arginine diets allowed for growth, weight gain, and meat creatine and arginine levels comparable to arginine‐sufficient treatments (Ale Saheb Fosoul et al., 2019; DeGroot et al., 2018, 2019; Dilger et al., 2013; Sharma et al., 2022; Yang et al., 2021), implying an arginine‐sparing effect. So far, this outcome of GAA consumption has not been assessed in humans. However, two interesting clinical trials demonstrated a significant increase in muscle size during GAA consumption in patients with poliomyelitis‐induced disability (Borsook et al., 1952), and neuromuscular disease (Aldes, 1957). Although arginine turnover has not been evaluated in these trials, an increase in muscle mass might be due to enhanced protein synthesis that utilizes surplus arginine spared by dietary GAA. A positive nitrogen balance suggested in another human trial with supplemental GAA (Borsook & Borsook, 1951b) might also indicate an increased arginine pool after GAA intake. Possible mechanisms involved in GAA‐driven improvements of protein synthesis/muscle growth might be due to several mechanisms that involve stimulating impact of GAA on insulin and insulin‐like growth factor‐1 secretion, activation of the mammalian target of rapamycin signaling pathway, upregulation of genes related to myogenesis, and downregulation of gene that encodes myostatin, a myokine that inhibits muscle cell growth and differentiation (for a detailed review, see Ostojic, Premusz, et al., 2020).

## 7 GAA AS TASTE MODULATOR?

GAA appears to possess a substantial effect on taste receptors. GAA exhibits a sweetness that is notably slow in onset relative to sucrose, particularly when evaluated at low levels of sweetness intensity (Nagarajan et al., 1996). GAA can activate T1R2 and T1R3 subsets of taste receptors (Nelson et al., 2001). The synthesis of three different disubstituted GAAs with intense sweet taste properties is reported (Sulikowski et al., 1995), with GAA analogs could be 200,000 times as sweet as sucrose (Hoffmann, 2008). Interestingly, GAA also enhanced the muscle flavor components of fish (Yang et al., 2021), but this effect might be associated with GAA‐driven regulation of fat metabolism (see below). Whether foods and dietary supplements rich in GAA affect the taste and food intake remains currently unknown. A possible GAA‐driven taste modulation might be another metabolic signal relevant to human nutrition, which requires further investigation.

## 8 DIETARY GAA AND FATTY LIVER

Non‐alcoholic fatty liver disease (NAFLD) is a complex chronic metabolic condition characterized by the deposition of fat in the liver that can lead to hepatic inflammation and organ failure. Although the NAFLD etiology is still under exploration, diet composition and quantity might be linked to disease pathogenesis and progression. NAFLD is often seen after diets deficient in methyl group donors, including choline and methionine (Radziejewska et al., 2020). Since GAA can act as a methyl group acceptor (Stead et al., 2001; Sugiyama et al., 1989), long‐term consumption of GAA might induce depletion of methyl groups in the liver and concomitant fat deposition. In a preliminary study, dietary GAA has been associated with fatty liver disease, where a 30‐day intake of GAA (1.2 g per kg feed) increased liver fat accumulation in rats (Baccari & Fidanza, 1947). Contrary to this historical study, a recent experimental report found that GAA treatment alone resulted in a histologically normal liver without evidence of hepatosteatosis (Osna et al., 2016). Still, GAA‐driven hepatic steatosis could be dose‐dependent, since the high doses administered in feeding studies (>4.5 g GAA per kg feed) were associated with higher GAA deposition and increased levels of homocysteine (demethylation product of methionine) in broilers and piglets liver (EFSA Panel on Additives and Products or Substances used in Animal Feed, 2016). The few data available allow the conclusion that homocysteine in muscle, liver, and kidney (as well as in plasma) will not increase up to GAA supplementation of 3.0 g per kg feed. For 6.0 g GAA per kg feed, a significant elevation of homocysteine in muscle, kidney, and plasma is described (EFSA Panel on Additives and Products or Substances used in Animal Feed, 2016). Human safety trials revealed no significant disturbances in biomarkers of liver damage after GAA consumption (Ostojic et al., 2013, 2018) yet no study with supplemental GAA assessed liver fat content using either invasive or non‐invasive techniques. Other methylation‐related side effects of dietary GAA exposure are reviewed in detail elsewhere (Ostojic, 2021), with GAA could also affect cellular uptake of thiamine (Zygmunt, 1984), another nutrient involved in adequate methylation in the liver.

## 9 CONCLUSION

**FIGURE 2:**
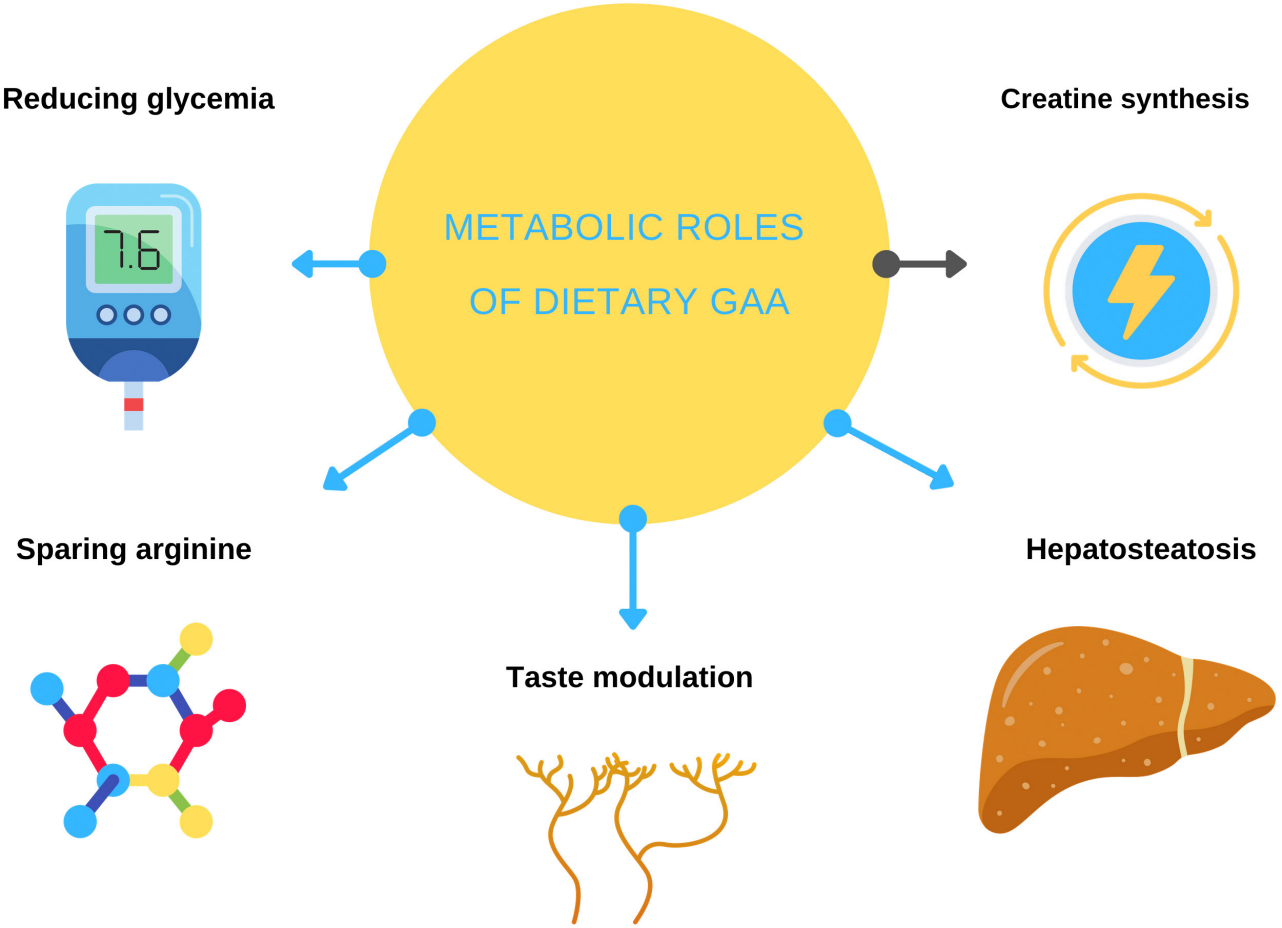
Possible metabolic roles of dietary GAA.

Besides acting as a direct precursor of creatine, dietary GAA is preliminary suggested to reduce blood glucose concentration, preserve arginine (both from the diet and produced internally) for other metabolic purposes, and possibly impact taste and/or food intake (Figure 2). Conditions that require dietary control of hyperglycemia or increase arginine demands (e.g., stress, rapid growth, wound healing) might thus benefit from adding GAA to a therapeutic dietary regimen. The above effects have to be accounted for the overall impact of GAA in human nutrition, along with possible organ‐specific consequences of GAA‐driven overconsumption of methyl group donors. Future studies are highly warranted to address the clinical relevance of the non‐creatine‐related impact of dietary GAA in normal and diseased populations. Immediate gaps that need to be dealt with include possible GAA‐driven hepatic steatosis and other methylation‐related effects of dietary GAA.

## ACKNOWLEDGEMENTS

None.

## FUNDING INFORMATION

No funding was received.

## CONFLICT OF INTEREST

SMO serves as a member of the Scientific Advisory Board on creatine in health and medicine (AlzChem LLC). SMO co‐owns patent “Supplements Based on Liquid Creatine” at European Patent Office (WO2019150323 A1), patent “Methods and Compositions for Improving a Response to a Metabolic Stress” at United States Patent and Trademark Office (US 2015/0150933 A1), and patent “Agent for Inhibiting Deterioration of Recognition Function Comprising Hydrogen Gas” at Japan Patent Office (ID 2016‐163,322). SMO has served as a speaker at Abbott Nutrition and has received research funding related to nutrition during the past 36 months from The World Health Organization, Serbian Ministry of Education, Science, and Technological Development, Provincial Secretariat for Higher Education and Scientific Research, Allied Beverages Adriatic, AlzChem GmbH, ThermoLife International, Hueston Hennigan LLP, HRW Natural Health Products Inc, Aktivátor Kft, and CarnoMed. SMO does not own stocks and shares in any organization. JJ declares no conflict of interest.

## ETHICS STATEMENT

Not applicable.

## Data Availability

No data has been used for this paper.
